# Triple Flat-Type Inductive-Based Oil Palm Fruit Maturity Sensor

**DOI:** 10.3390/s18082496

**Published:** 2018-08-01

**Authors:** Nor Aziana Aliteh, Norhisam Misron, Ishak Aris, Roslina Mohd Sidek, Kunihisa Tashiro, Hiroyuki Wakiwaka

**Affiliations:** 1 Faculty of Engineering, Universiti Putra Malaysia, Serdang, Selangor 43400, Malaysia; ishak_ar@upm.edu.my (I.A.); roslinams@upm.edu.my (R.M.S.); 2 Institute of Advance Technology (ITMA), Universiti Putra Malaysia, Serdang, Selangor 43400, Malaysia; 3 Faculty of Engineering, Shinshu University, Wakasato 4-17-1, Nagano 380-8553, Japan; tashiro@shinshu-u.ac.jp (K.T.); wakiwak@shinshu-u.ac.jp (H.W.)

**Keywords:** inductive concept, air coil, resonance frequency, oil palm, maturity classification, moisture content

## Abstract

This paper aims to study a triple flat-type air coil inductive sensor that can identify two maturity stages of oil palm fruits, ripe and unripe, based on the resonance frequency and fruitlet capacitance changes. There are two types of triple structure that have been tested, namely Triple I and II. Triple I is a triple series coil with a fixed number of turns (*n* = 200) with different length, and Triple II is a coil with fixed length (*l* = 5 mm) and a different number of turns. The peak comparison between Triple I and II is using the coefficient of variation $c_{v}$, which is defined as the ratio of the standard deviation to the mean to express the precision and repeatability of data. As the fruit ripens, the resonance frequency peaks from an inductance–frequency curve and shifts closer to the peak curve of the air, and the fruitlet capacitance decreases. The coefficient of the variation of the inductive oil palm fruit sensor shows that Triple I is smaller and more consistent in comparison with Triple II, for both resonance frequency and fruitlet capacitance. The development of this sensor proves the capability of an inductive element such as a coil, to be used as a sensor so as to determine the ripeness of the oil palm fresh fruit bunch sample.

## 1. Introduction

The year 2017 marked the 100 year anniversary of Malaysia’s oil palm industry, after Henri Fauconnier began to commercialize the oil palm plantation at the Tennamaram Plantation, Selangor, in 1917. Malaysia currently accounts for 39% of the world’s palm oil production and 44% of the world’s exports. Therefore, Malaysia has an important role, as one of the biggest palm oil products’ producers and exporters, in fulfilling the growing sustainable global need for oils and fats [1]. The oil palm tree is well-known as one of the most efficient oilseed crops in the world. One hectare of an oil palm plantation is able to harvest up to ten times more than the other oilseed crops [2]. The *Elaeis guineensis* is the most common species of oil palm in the oil palm plantation because of its thick mesocarp and thin endocarp, making it suitable for commercialization [3]. The oil palm fresh fruit bunch (FFB) will undergo a crude palm milling process to extract the palm oil as well as the palm oil by-products. The standard procedure to grade the oil palm procedure is commonly done through visual inspection by human graders, based on the oil palm grading manual that was published by the Malaysian Palm Oil Board (MPOB). The ripeness of the oil palm FFB is identified primarily from the colour of the oil palm fruit exocarp, as well as from the number of loose fruit or empty sockets in the bunch [4]. Furthermore, it is important to pluck the oil palm FFB at the optimal maturity stages in order to maximize the rate of extraction. Various methods for oil palm fruit maturity grading and detection methods have been introduced. The most popular method is the spectroscopy method with Red-Green-Blue (RGB) visual imaging techniques and software analysis [5,6,7,8,9,10]. The laser-based imaging ripeness detection method [11] has also been introduced, as well as using the fluorescent technique [12]. M. Saufi et al. [13] introduced an oil palm fruit grading using Near Infrared (NIR) imaging and D. Silalahi et al. [14] presented the Genetic Algorithm Neural Network (GANN) software to analyse the NIR spectral data. In addition, S. Zolfagharnassab et al. [15] initiated a comparison using a thermal sensor to detect changes in the mean temperature as the oil palm FFB ripens. Moreover, S. Shaarani et al. [16] proposed an oil palm fruit ripeness monitoring development with the use of magnetic resonance imaging (MRI), together with bulk nuclear magnetic resonance (NMR). Besides that, the microwave moisture sensor was introduced for an in-situ measurement of the complex permittivity of moisture content, using a six- and five-port reflectometer [17,18,19]. This study is a continuation on the work of N. Hasmiza et al., who introduced a new inductive concept using a circular coil [20], single flat-type air coil with various dimensions [21], dual resonance frequency effect [22], and relative water content of oil palm FFB using single flat-type air coil was also estimated against week [23]. This paper aims to develop a triple series flat-type air coil structure with a triple resonance peak to test the effectiveness of detecting the ripeness of the selected oil palm FFB, based on the increase in the effectiveness of a dual resonance frequency, when compared to the single coil [22], but with a weekly field data analysis that was similar to the relative water content method [23].

## 2. Basic Concept of Detection and Methodology

**Figure 1:**
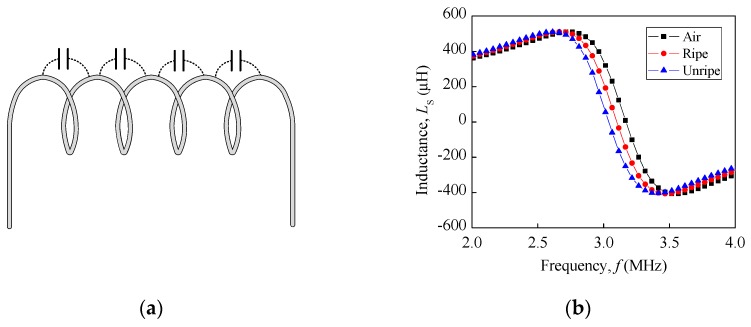
(**a**) Self-capacitance between coil turn and (**b**) oil palm fruit ripeness for *L*_s_–*f* curve for single-flat type air coil for air, ripe, and unripe.

The inductor is generally a passive element that stores energy in the form of a magnetic field. The basic detection concept in this study relies on the behaviour of a non-ideal inductor at a high frequency. Other than the resistive component in a non-ideal inductor, there is also capacitive effect that affects the inductance property of an inductor, as shown in Figure 1a. The self-capacitance is significant at a high frequency, which vastly depends on the coil’s turn-to-turn effect. The presence of a tiny capacitance between the winding is because of the coil’s wire insulated coating and each of the winding sections that considerably have a different potential, as a result of their own inductance and resistance. The graph that was obtained through the measurement is shown in Figure 1b, with an inductance–frequency (*L_s_–f*) curve for air, ripe, and unripe fruit for the single flat-type air coil similar to [23]. It is shown that the unripe, ripe, and air follow the same sequence, where the unripe fruit has the lowest resonance frequency, while the air has the highest peak resonance frequency. As the fruit ripens, the resonance curve increases and shifts toward the air peak resonance frequency.

**Figure 2:**
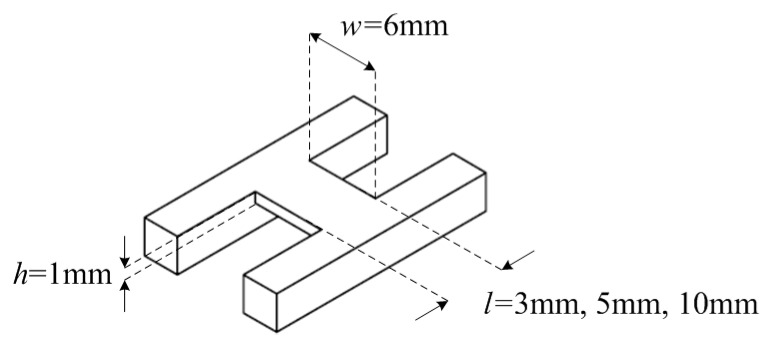
Air coil sensor structure with its design specification.

The H-shaped air coil core as shown in Figure 2 makes it easy to wind the coil manually. The design is also made to have a flat surface on one side for the coil’s maximum contact with the fruitlet flesh. The width *w* and height *h* is kept constant at 6mm and 1mm, respectively, whereas the length is varied according to the design specification, as shown in Table 1. Triple I has a constant number of turns, *n* = 200, and Triple II has constant length, *l* = 5 mm. The first, second, and third coil series configuration for both Triple I and II were arranged with increasing inductance, where the first coil had the largest inductance and the third coil had the smallest inductance.

**Figure 3:**
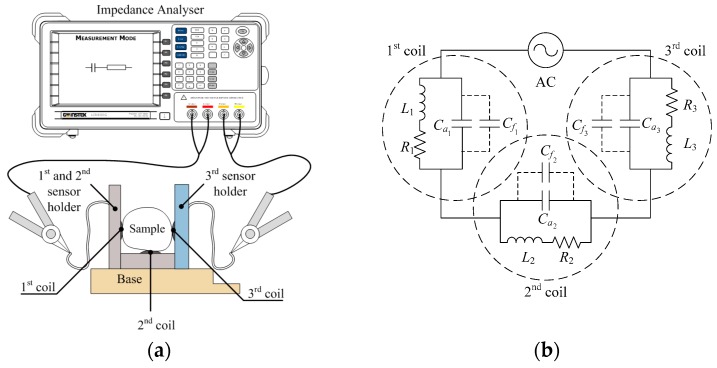
(**a**) Experiment setup for triple flat-type air coil sensor and its (**b**) equivalent circuit.

The experiment setup for triple flat-type air coil is shown in Figure 3a. The triple coil sensor was directly connected to the impedance analyser with the setup parameter, as presented in Table 2, which remained constant throughout the experiment. The basic circuit representation for Figure 3a is as shown in Figure 3b, where the inductance ($L_{1}$, $L_{2}$, and $L_{3}$), internal resistance ($R_{1}$, $R_{2}$, and $R_{3}$), and the total self-capacitance, which was composed of air ($C_{a_{1}}$, $C_{a_{2}}$, and $C_{a_{3}}$) and fruitlet capacitance ($C_{f_{1}}$, $C_{f_{2}}$, and $C_{f_{3}}$) in parallel. The dotted line indicates the assumed to be fruitlet capacitance that came from the oil palm fruitlet sample.

**Figure 4:**
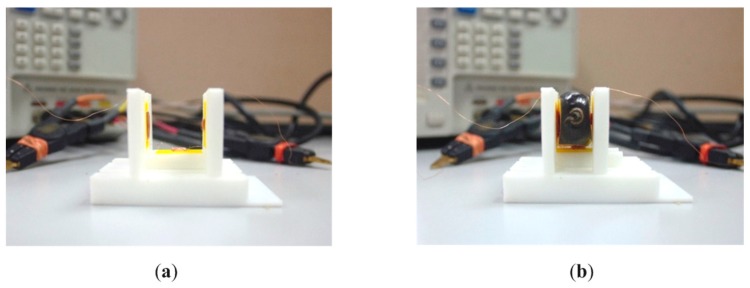
Triple flat-type air coil sensor (**a**) without and (**b**) with sample.

Figure 4a,b shows the actual experiment setup without and with the sample. Weekly fruitlet samples were collected and measured using an impedance analyser. Every week before the experiment was conducted, the coil sensor needed to be measured without any sample to be used as a reference, as shown in Figure 4a, in order to eliminate any measurement error because of the changing value of the resonance frequency over time. Figure 4b shows an important data collection step, where the fruitlet was sliced into three flat surfaces for the flesh to touch the coil, in order to obtain the maximum detection.

## 3. Experiment Analysis

### 3.1. Self-Capacitance and Fruitlet Capacitance

**Figure 5:**
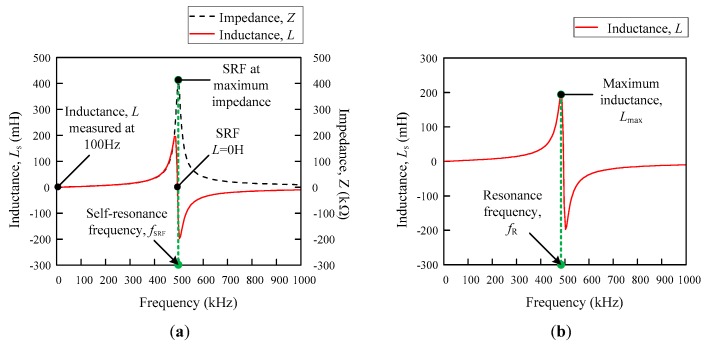
*L_s_–f* curve illustrates (**a**) self-resonance frequency (SRF) and (**b**) resonance frequency.

The value of self-capacitance can be estimated using the general resonance frequency formula as follows:

(1) $$f_{R}=\frac{1}{2\pi \sqrt{LC}},$$

where $f_{R}$ (Hz) is the resonance frequency, *L* (H) is the inductance, and *C* (F) is the capacitance. There are two different resonances principles that are present in this research, the self-resonance frequency (SRF) and resonance frequency, which was obtained through the maximum peak in the inductance–frequency (*L_s_–f*) curve from the impedance analyser. Both of the resonances used the same formula as shown in Equation (1), but had a different value of the inductance, *L*, and resonance frequency, $f_{R}$. Figure 5a,b illustrate the differences between them. The SRF is the resonance frequency that occurs at $L_{s}$ = 0 H and the standard value of the inductance that is used is measured at 100 Hz [24]. On the other hand, the resonance frequency that is used for the analysis in this study is referred to as the maximum inductance peak of the *L_s_–f* curve, as shown in Figure 4b. From this information, the self-capacitance can be estimated from both methods, but the approach that has been used throughout this research is based on the information that was gained from Figure 5b.

The self-capacitance and fruitlet capacitance calculation are rather straightforward from Equation (1), and the parameter taken is, as shown in Figure 4b, and rearranged as follows:

(2) $$C_{R}=\frac{1}{L_{max}}\cdot \frac{1}{{\left(2\pi f_{R}\right)}^{2}},$$

where the $C_{R}$ (F) is the calculated by the capacitance at the resonance, by substituting the maximum peak inductance, $L_{max}$ (H) and the resonance frequency $f_{R}$ (Hz) at $L_{max}$. The fruitlet capacitance $C_{f}$ is determined from equation below, as follows:

(3) $$C_{f}=C_{R}-C_{a},\;$$

where $C_{f}$ (F) is the fruitlet capacitance, $C_{R}$ (F) is the total self-capacitance that is obtained through Equation (2), and $C_{a}$ (F) is the capacitance that is calculated using the measured resonance frequency of air, with no sample, it was considered measuring the air literally. The capacitance at the peak resonance frequency $C_{R}$ is introduced especially so as to avoid confusion with the total self-capacitance that can be obtained at any given frequency using Equation (1).

### 3.2. Comparison Analysis Method

**Figure 6:**
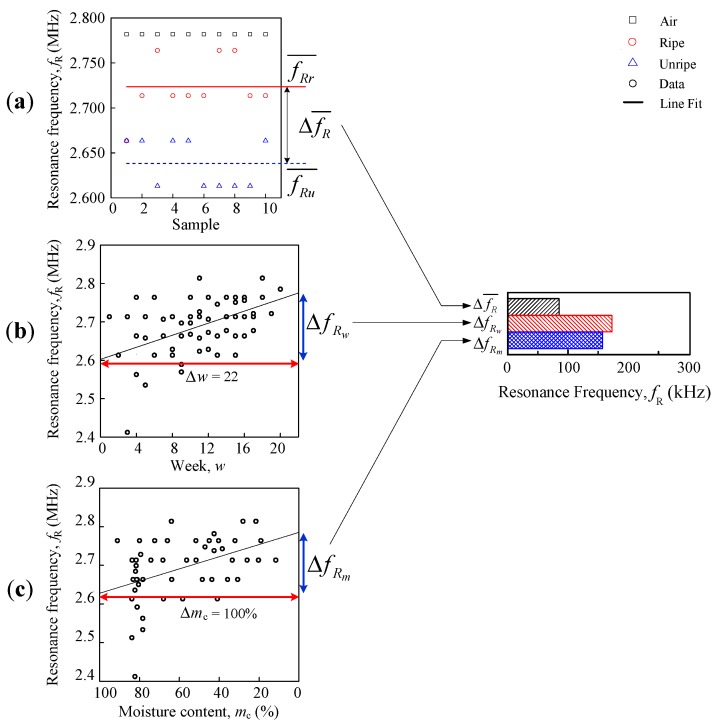
Resonance frequency for (**a**) direct ripe-unripe comparison, (**b**) resonance frequency against the weeks and (**c**) resonance frequency against moisture comparison for selected flat-type air coil peak.

The comparison analyses are divided into three, ripe-unripe direct comparison, comparison against a week, and moisture content, as shown in Figure 6. The differences are further summarized into a horizontal bar graph. There were two types of data sets that were used for the analysis in this paper, resonance frequency and fruitlet capacitance.

The first evaluation used is direct ripe-unripe comparison analysis as illustrated in Figure 6a where the mean difference for peak resonance frequency $\Delta \bar{f_{R}}$ (Hz) and fruitlet capacitance $\Delta \bar{C_{f}}$ (F) are calculated using formula:

(4) $$\Delta \bar{f_{R}}=\bar{f_{Rr}}-\bar{f_{Ru}},$$

(5) $$\Delta \bar{C_{f}}=\Delta \bar{C_{fr}}-\Delta \bar{C_{fu}},$$

where $\Delta \bar{f_{Rr}}$ (Hz) is mean ripe resonance frequency, $\Delta \bar{f_{Ru}}$ (Hz) is the mean unripe resonance frequency, $\bar{C_{f_{r}}}$ (F) is the mean ripe fruitlet capacitance, and $\bar{C_{f_{u}}}$ (F) is the mean unripe fruitlet capacitance.

The approximation regression line is fit for a week, and the moisture content evaluation for both the coil resonance frequency and the fruitlet capacitance are shown in Figure 6b,c, respectively, which follows the general line equation below, as follows:

(6) $$y=\alpha +\beta x\;\mathrm{where}\;\beta =\frac{\Delta y}{\Delta x}\;$$

where *y* is the *y*-axis component, such as the resonance frequency or fruitlet capacitance; and *x* is the *x*-axis component, either the weeks or the moisture content according to the graph. The β value is the sensitivity of the coil sensor.

For the second evaluation analysis against a week, the common notation for both the resonance frequency $f_{R}$ (Hz) and the fruitlet capacitance $C_{f}$ (F) data setsis as follows: *w* is the number of weeks and Δw is fixed at 22 weeks. Equation (6) has been further defined for the resonance frequency and fruitlet capacitance against the week, as follows:

(7) $$f_{R}=\alpha_{wf_{R}}+\left(\beta_{wf_{R}}\cdot \;w\right)\;$$

(8) $$\beta_{wf_{R}}=\frac{\Delta f_{R_{w}}}{\Delta w}=\frac{\Delta f_{R_{w}}}{22}\;$$

(9) $$\Delta f_{R_{w}}=\beta_{wf_{R}}\cdot 22\;$$

where $\alpha_{wf_{R}}$ (Hz) is the frequency at *w* = 0 on the resonance frequency against the weeks graph, $\beta_{wf_{R}}$ (Hz/week) is the sensitivity of the coil sensor resonance frequency with respect to the week, and $\Delta f_{R_{w}}$ (Hz) is the resonance frequency of the weeks difference.

(10) $$C_{f}=\alpha_{wC_{f}}+\left(\beta_{wC_{f}}\cdot \;w\right)\;$$

(11) $$\beta_{wC_{f}}=\frac{\Delta C_{f_{w}}}{\Delta w}=\frac{\Delta C_{f_{w}}}{22}\;$$

(12) $$\Delta C_{f_{w}}=\beta_{wC_{f}}\cdot 22\;$$

where $\alpha_{wC_{f}}$ (F) is the fruitlet capacitance at *w* = 0 on the fruitlet capacitance against the weeks graph, $\beta_{wC_{f}}$ (F/week) is the sensitivity of the coil sensor of the fruitlet capacitance with respect to the week, and ${\Delta C}_{f_{w}}$ (F) is the fruitlet capacitance of the weeks difference.

Furthermore, for the third evaluation analysis against the moisture content, the common notations that were used for both sets of data are as follows: $m_{c}$ (%) is the moisture content in percentage and $\Delta m_{c}$ is fixed at 100%. Hence, the equation for the resonance frequency $f_{R}$ (Hz) and fruitlet capacitance $C_{f}$ (F) against the moisture content are as follows:

(13) $$f_{R}=\alpha_{mf_{R}}+\left(\beta_{mf_{R}}\cdot m_{c}\right)\;$$

(14) $$\beta_{mf_{R}}=\frac{\Delta f_{R_{m}}}{\Delta m_{c}}=\frac{\Delta f_{R_{m}}}{100\%}\;$$

(15) $$\Delta f_{R_{m}}=\beta_{mf_{R}}\cdot 100\%\;$$

where $\alpha_{mf_{R}}$ (Hz) is the resonance frequency at $m_{c}$ = 0% on the resonance frequency against the moisture graph, $\beta_{mf_{R}}$ (Hz/%) is the sensitivity of the coil sensor with respect to moisture content, $\Delta f_{R_{m}}$ (Hz) is the resonance frequency moisture content difference.

(16) $$C_{f}=\alpha_{mC_{f}}+\left(\beta_{mC_{f}}\cdot m_{c}\right)\;$$

(17) $$\beta_{mC_{f}}=\frac{\Delta C_{f_{m}}}{\Delta m_{c}}=\frac{\Delta C_{f_{m}}}{100}\;$$

(18) $$\Delta C_{f_{m}}=\beta_{mC_{f}}\cdot 100\%\;$$

where $\alpha_{mC_{f}}$ (F) is the fruitlet capacitance at $m_{c}$ = 0% on the fruitlet capacitance against the moisture graph, $\beta_{mC_{f}}$ (F/%) is the sensitivity of the coil sensor with respect to moisture content, and ${\Delta C}_{f_{m}}$ (Hz) is the fruitlet capacitance moisture content difference. Note that the resonance frequency against the moisture content begins with 100%, in Figure 6c, with the purpose of following the time vector (week) pattern so as to observe its trend and therefore, its gradient value is actually negative when compared to the weeks graph in Figure 6b.

Further analysis was conducted on the data in order to compare the ripe–unripe, week, and moisture differences. A simple statistical method was introduced to observe the variability and stability of the data. In order for the coil configuration to be selected, the coil needs to have a small variability as well as a high output sensitivity for the best performance.

Differences in the mean $\bar{\Delta }$ and standard deviation σ were introduced as well as the coefficient of variation $c_{v}$, to compare both of the triple flat-type air coil performances. The coefficient of the variation $c_{v}$ is a standardized measure of dispersion, which is defined as the ratio of the standard deviation to the mean. $c_{v}$ is widely used to express the precision and repeatability of the data [25].

(19) $$c_{v}=\frac{\sigma }{\bar{\Delta }}\;$$

where *σ* is the standard deviation and $\bar{\Delta }$ is the average of the differences for the resonance frequency and fruitlet capacitance, as shown in Equations (20) and (21), respectively.

(20) $$\bar{\Delta }f_{R}=\frac{\Delta \bar{f_{R}}+\Delta f_{R_{w}}+\Delta f_{R_{m}}}{3}\;$$

(21) $$\bar{\Delta }C_{f}=\frac{\Delta \bar{C_{f}}+{\Delta C}_{f_{w}}+\Delta C_{f_{m}}}{3}\;$$

where $\bar{\Delta }f_{R}$ (Hz) is the resonance frequency differences mean, which consist of $\Delta \bar{f_{R}}$, $\Delta f_{R_{w}}$ and $\Delta f_{R_{m}}$ from Equations (4), (9), and (15), respectively. Whereas $\bar{\Delta }C_{f}$ (F) is the fruitlet capacitance differences mean that consist of $\Delta \bar{C_{f}}$, ${\Delta C}_{f_{w}}$, and $\Delta C_{f_{m}}$ from Equations (5), (12), and (18), respectively. For a comparison between the data sets with different means, the coefficient of the variation is preferred instead of the standard deviation. Since the value of $c_{v}$ is a dimensionless number independent of the unit in which the measurement is calculated, the sensor needed to be designed so that the coefficient of the variation $c_{v}$ was close to zero, where the data yields a constant absolute error over the operational range.

## 4. Results and Discussions

### 4.1. Sample Selection and Bunch Moisture Content

**Figure 7:**
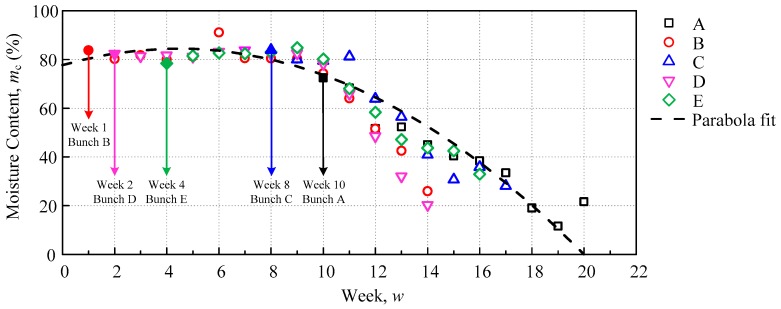
The moisture content for fruit sample bunch A, B, C, D, and E.

The oil palm tree (*Elaeis guineensis*) with the variety named *Tenera*, which came from hybridization of *Dura* and *Pisifera*, was selected for this study. Five selected bunches were tested every week until the oil palm FFB ripened. The oil palm trees that were involved in this experiment were approximately 6 years old and 3 m in height. A total of 5 fruitlet samples were taken weekly from the selected bunches and a total of 66 fruitlet samples throughout the experiment period, until each bunch ripened. The samples were then measured with an impedance analyser. After the measurement, the fruitlet moisture content was determined using an oven-drying method. The sample was sliced and dried in the oven at 103 °C ± 2 °C until the weight of the sample became constant. Based on the moisture content of the fruitlet, the fruitlet age was approximated, as shown in Figure 7, where samples A, B, C, D, and E were assumed to be at weeks 10, 1, 8, 2, and 4, respectively, hence, the moisture content estimation equation from Figure 7 is as follows:

(22) $$m_{c}=3.348w^{2}+3.075w+77.67,\;\mathrm{For}\;\;0<w<20\;$$

where $m_{c}$ (%) is the moisture content and *w* is the number of weeks. The parabolic fit has coefficient of determination, $R^{2}=0.89367$, where the estimation is valid for the range, begins at week 1, which was deduced to be at 80.4%, and week 19, which was deduced to be at 10.47%. The over-ripe fruit after week 19 was predicted to be at a constant ripe percentage, around 10% to 40% and did not go below 10%.

### 4.2. Inductance-Frequency Graph Characteristics

**Figure 8:**
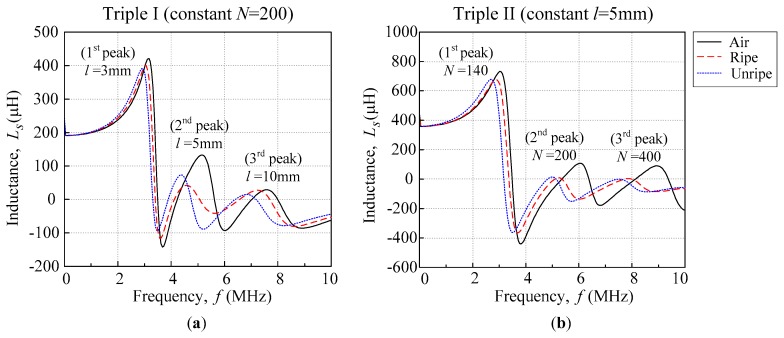
(**a**) Triple I and (**b**) Triple II *L*_s_–*f* curves for air, ripe, and unripe conditions.

Figure 8a,b below shows the behaviour of the triple resonance of the air, ripe, and unripe for Triple I and II, which followed the sequence that was similar to its single flat-type air coil result in Figure 1b. Triple II was observed to have had a higher first maximum inductance peak when compared with Triple I. However, the Triple II second and third peak were shorter compared with the Triple I second and third peak. The second and third peak trend affected the peak detection and caused the data to be unstable. Therefore, the first peak performance was the important parameter in order to select the best performance indicator. This observation was supported by the previous study by N. Hasmiza et al. [22], who mentioned that the first peak of the dual coil that was constructed was dominating the test results. It was observed that the behaviour of the dual and triple coil configuration was similar in nature, with its maximum peak inductance decreasing when the inductance of the coil decreased.

### 4.3. Peak Resonance Frequency, $f_{R}$

This section examines the peak resonance frequency differences between the ripe–unripe sample comparison, as well as the weeks and moisture content evaluation of the oil palm fruitlet sample for the triple flat-type air coil.

**Figure 9:**
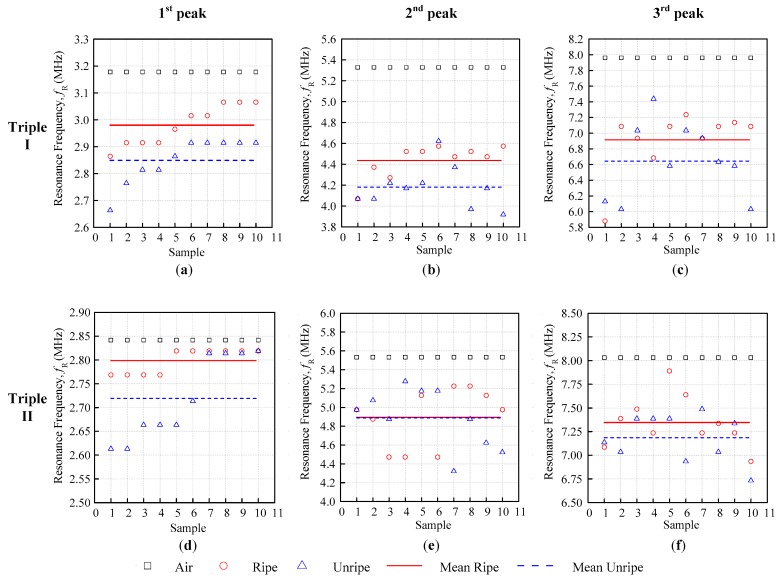
Ripe-unripe first, second, and third peak resonance frequency comparison for Triple I (**a**–**c**) and Triple II (**d**–**f**).

Firstly, the ripe-unripe sample comparison is summarized in Table 3, and the mean value was obtained from Figure 9a–f, using Equation (4), for Triple I and II when calculating the resonance frequency mean difference $\Delta \bar{f_{R}}$. The ripe-unripe comparison result showed that the difference between $\bar{f_{Rr}}$ and $\bar{f_{Ru}}$ decreased as the inductance of the coil increased, except for the Triple II second peak with a small mean difference $\Delta \bar{f_{R}}$ = 5.03 kHz. When comparing Triple I and II, it seemed that Triple I had a bigger $\Delta \bar{f_{R}}$ value for all of the peaks when compared with Triple II. Furthermore, even though the coil configuration for the second peak was the same, the performance differed greatly, as the Triple I and Triple II $\Delta \bar{f_{R}}$ values were 256.28 kHz and 5.03 kHz, respectively.

**Figure 10:**
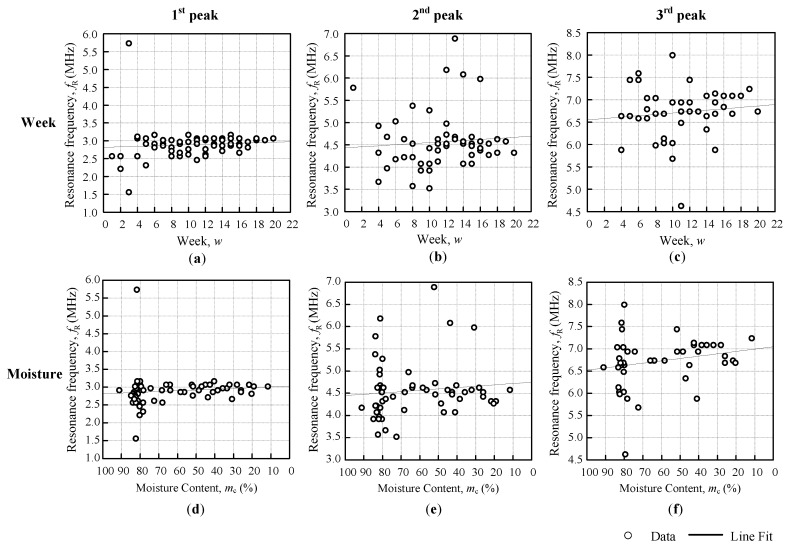
Triple I first, second, and third peak resonance frequency against the weeks (**a**–**c**) and moisture (**d**–**f**).

**Figure 11:**
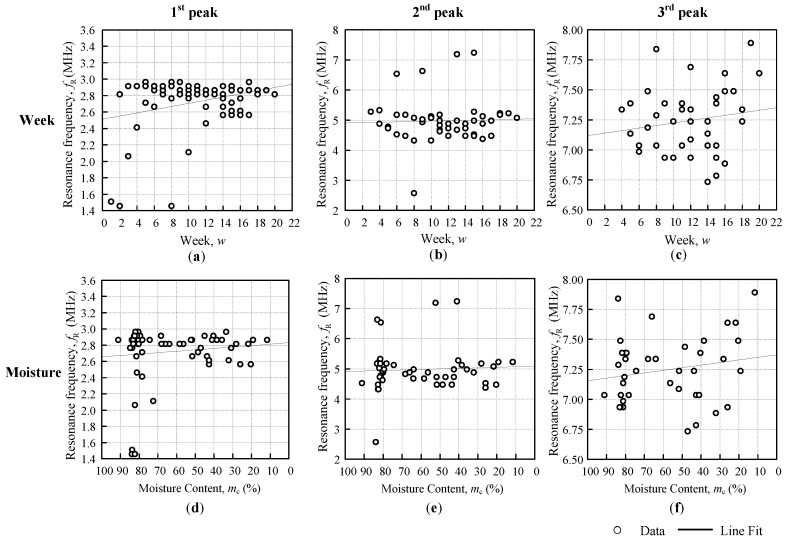
Triple II first, second, and third peak resonance frequency against the weeks (**a**–**c**) and moisture (**d**–**f**).

Figure 10 and Figure 11 shows the resonance frequency $f_{R}$ against the weeks and moisture content graph, with a line fit that followed the general approximate regression from Equation (5). The Triple I and II line fits that were obtained from Figure 9 for the Triple I peaks, and Figure 10 for the Triple II peaks, are summarized in Table 4. The linear regression equation for $f_{R}$ against the weeks was based on Equation (6), and the linear regression equation $f_{R}$ against the moisture was based on Equation (9). The resonance frequency against the weeks showed all of the positive gradients $\beta_{wf_{R}}$, but the moisture content of the fruitlet was inversely proportional to the resonance frequency, therefore producing a negative gradient $\beta_{mf_{R}}$. From Table 4, it was observed that the Triple I sensitivity increased with the decreasing inductance against both the weeks and moisture content. Triple II $f_{R}$ against the weeks sensitivity $\beta_{wf_{R}}$ was rather inconsistent, but for $f_{R}$ against the moisture sensitivity $\beta_{mf_{R}}$, magnitude increased slightly with the increasing coil inductance.

**Figure 12:**
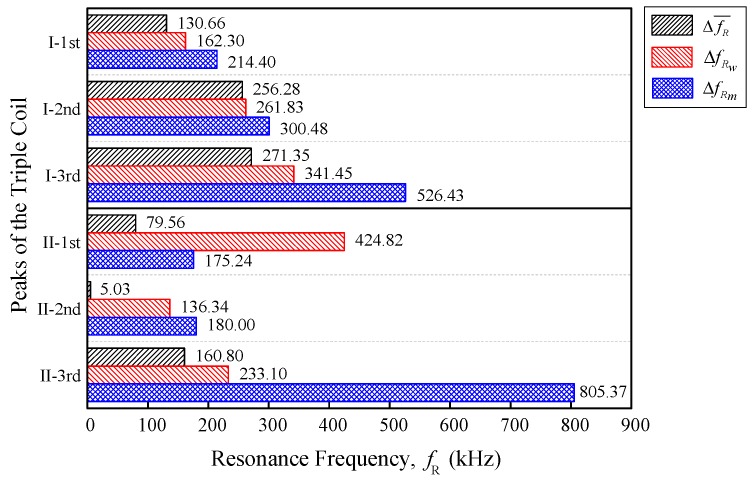
Triple I and II resonance frequency evaluation comparison for $\Delta \bar{f_{R}}$, $\Delta f_{R_{w}}$, and $\Delta f_{R_{m}}$.

The resonance frequency evaluation comparison between the peaks of Triple I and II are summarized in Figure 12, below. There were three parameters that were compared,$\Delta \bar{f_{R}}$, $\Delta f_{R_{w}}$, and $\Delta f_{R_{m}}$. Equation (4) was used to calculate the resonance frequency mean difference $\Delta \bar{f_{R}}$ and Table 3 summarizes the data from Figure 9. The value of $\Delta f_{R_{w}}$ was obtained through $\beta_{wf_{R}}$, using Equation (8), with Δw being fixed at 22 from the linear regression equation for $f_{R}$ against the week. The linear regression equation for $f_{R}$, against the moisture were based on Equation (13), with the value of the moisture $\Delta f_{R}$ being obtained through the gradient $\beta_{mf_{R}}$, using Equations (14) and (15) with $\Delta m_{c}$ = 100%.

Table 5 summarizes all of the differences from Figure 12 with the differences mean, standard deviation, and coefficient of the variation $c_{v}$. When comparing the first peak of Triple I and II, the Triple I had a smaller difference mean $\bar{\Delta }f_{R}$ = 169.12 kHz in comparison with Triple II $\bar{\Delta }f_{R}$ = 226.54 kHz. For the second peak, even though the coil configuration for both of the triple series were the same, it seemed that Triple I had a higher $\bar{\Delta }f_{R}$, as compared with Triple II by 165.74 kHz. The third peak comparison showed that the Triple I was slightly lower than the Triple II third peak by 20.02 kHz.

The dual coil structure of with a different number of turns (200–140), which was researched by N. Hasmiza et al. [22], pointed out a noticeable 371% improvement in terms of the difference between the sample’s ripe and unripe mean for both of the samples when comparing them with the single flat-type air coil with the same coil structure and number of turns, *n* = 200 [21]. Even though it was a comparison of normalized difference, the idea of the mean ripe minus the mean unripe, which was similar to the differences mean $\bar{\Delta }f_{R}$, was used for this comparison. Using $\bar{\Delta }f_{R}$ to compare the first peak of Triple I and II, the differences mean for first peak of Triple I was smaller by 75%, compared with Triple II, which meant that the Triple II sensitivity was better than that of Triple I. It was noted that the $\bar{\Delta }f_{R}$ was the average of $\Delta \bar{f_{R}}$, $\Delta f_{R_{w}}$, and $\Delta f_{R_{m}}$. However, in terms of the precision of its variation across the three types of evaluations that were tested, it was observed that Triple I had a more consistent value in comparison with Triple II.

### 4.4. Fruitlet Capacitance, $C_{f}$

This section studied the fruitlet capacitance differences between the ripe–unripe sample comparison, in addition to the weeks and moisture content evaluation of the oil palm fruitlet sample for the triple flat-type air coil.

**Figure 13:**
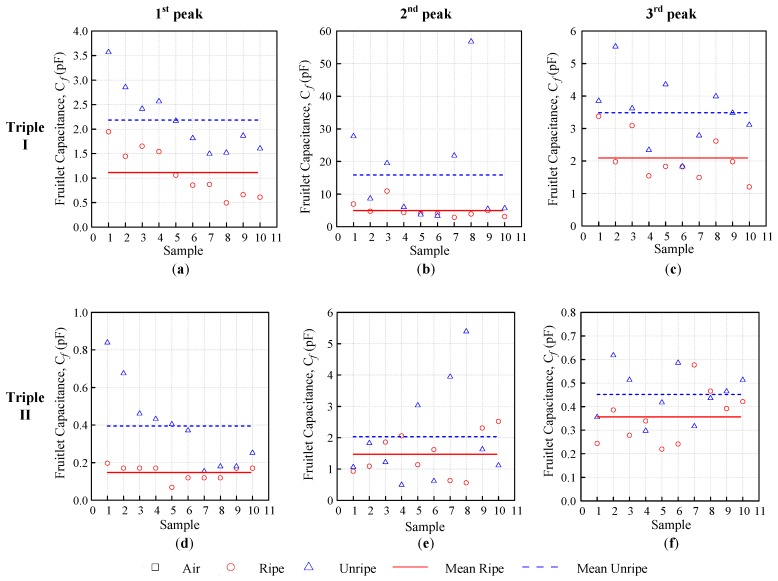
Ripe-unripe first, second, and third fruitlet capacitance comparison for Triple I: (**a**–**c**) and Triple II: (**d**–**f**).

The fruitlet capacitance $C_{f}$ was acquired when the self-capacitance of the air coil $C_{a}$ was deducted from the self-capacitance of the coil with the fruitlet sample $C_{R}$, as expressed in Equation (3). In contrast to the ripe resonance frequency mean $\bar{f_{Rr}}$ and the unripe resonance frequency mean $\bar{f_{Ru}}$, the mean unripe fruitlet capacitance $\bar{C_{fu}}$ was bigger than the mean ripe $\bar{C_{fr}}$, as demonstrated in Figure 13. The fruitlet capacitance mean difference $\Delta \;\bar{C_{f}}$ was obtained through Equation (5) and is tabulated in Table 6. The overall fruitlet capacitance mean for the ripe and unripe for Triple I was also relatively higher than Triple II, when compared peak-to-peak. The difference $\Delta \;\bar{C_{f}}$ for Triple I was also higher than Triple II. However, the Triple I second peak had shown incredibly big differences, as compared to the rest of the peaks, for both Triple I and II with $\Delta \;\bar{C_{f}}$ = 10.872 pF. Even though Triple II had the same second coil configuration as Triple I, the value of $\bar{C_{fr}}$, $\bar{C_{fu}}$, and the difference $\Delta \;\bar{C_{f}}$ was not as high as in the Triple I configuration.

**Figure 14:**
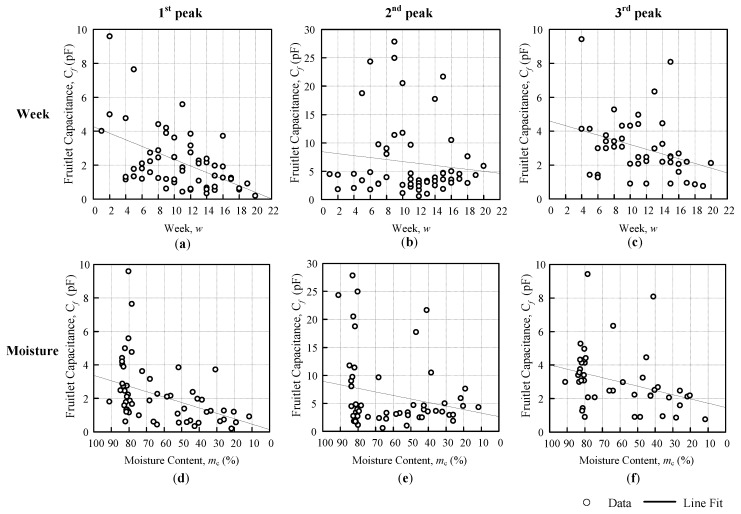
Triple I first, second, and third fruitlet capacitance against the weeks (**a**–**c**) and moisture content (**d**–**f**).

**Figure 15:**
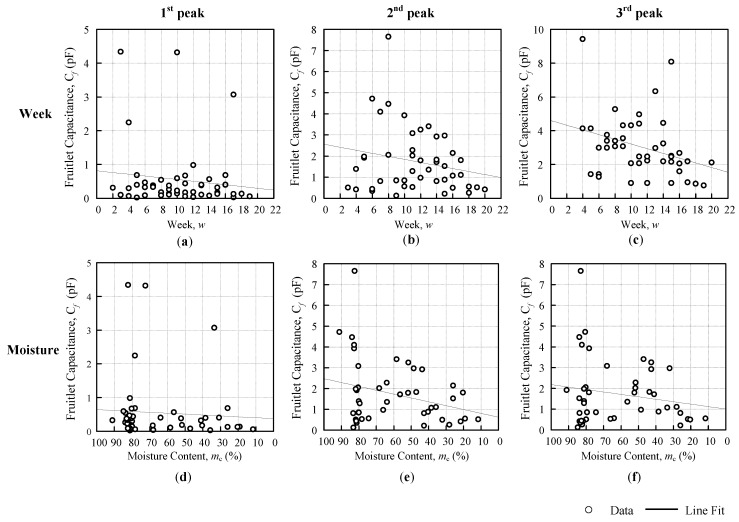
Triple II 1st, 2nd and 3rd fruitlet capacitance against the weeks (**a**–**c**) and moisture (**d**–**f**).

The individual fruitlet capacitance against the weeks and moisture content graph is presented in Figure 14 for Triple I, and in Figure 15 for Triple II. The line fit equation parameter value from Figure 14 and Figure 15 are summarized in Table 7, below. Both of the figures are separated by the peaks, namely, the first, second, and third with the fruitlet capacitance against the weeks and moisture. The linear regression equation was defined from Equations (13) and (16) for both the weeks and moisture graph. From Table 7, the $\alpha_{wC_{f}}$ value for the $C_{f}$ against the weeks, showed an estimation for the unripe fruitlet capacitance, whereas the $\alpha_{mC_{f}}$ value of the $C_{f}$ against the moisture content showed the fruitlet capacitance of the air, for which the that was value observed was close to zero. The $C_{f}$ against the weeks’ gradient showed all of the negative $\beta_{mC_{f}}$, but the $C_{f}$ against the moisture content showed a positive gradient $\beta_{mC_{f}}$. It was because, as the moisture content decreased, the fruitlet capacitance decreased as well. This result trend was similar to the result that was shown by K. Y. Lee et al. [18], where the dielectric constant increased with increasing moisture content.

**Figure 16:**
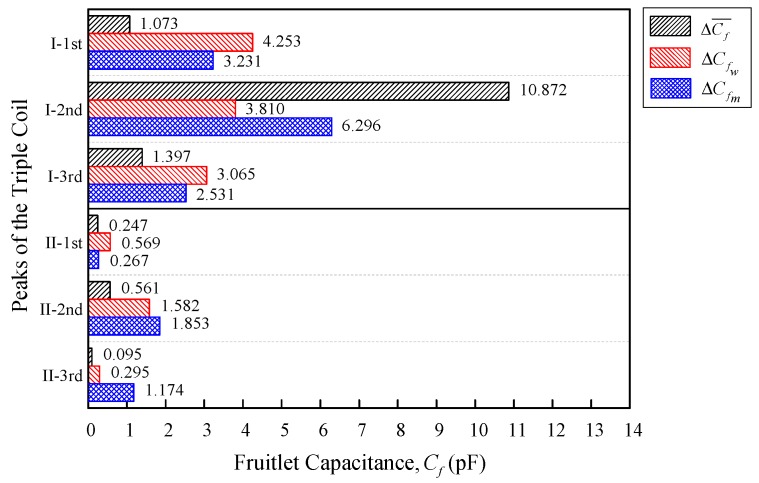
Triple I and II fruitlet capacitance evaluation comparison for $\Delta \bar{C_{f}}$, ${\Delta C}_{f_{w}}$, and $\Delta C_{f_{m}}$.

Figure 16 summarizes the fruitlet capacitance comparison between the peaks of Triple I and II. There were three parameters that were compared, namely, $\Delta \;\bar{C_{f}}$ and $\Delta C_{f}$ for the weeks and moisture, respectively. The fruitlet capacitance mean difference $\Delta \;\bar{C_{f}}$ was obtained through Equation (5) and is tabulated in Table 6. Furthermore, for the triple series comparison between the peaks in Figure 16, Equation (12) with Δw = 22 was used to evaluate ${\Delta C}_{f_{w}}$, whereas Equation (18) with $\Delta m_{c}$ = 100% was used to evaluate $\Delta C_{f_{m}}$.

Table 8 summarizes all of the differences from Figure 16, with the differences mean, standard deviation, and coefficient of variation $c_{v}$. Relatively, Triple I had a higher fruitlet capacitance differences mean $\bar{\Delta }C_{f}$, as compared to Triple II. When comparing the second peak of both of the triple series, both had performed with the highest $\bar{\Delta }C_{f}$, but the Triple I second peak was 5.25 times bigger compared with the Triple II second peak, even though both had the same *n* = 200 and *l* = 5 mm coil configuration. Moreover, the value of the coefficient of the variation $c_{v}$ peak-to-peak comparison between the Triple I and II peaks showed that all $c_{v}$ of Triple I were smaller than Triple II. The big value of all of the $c_{v}$ means that were shown by the Triple II third peak indicated that it had an inconsistent result for all three of the evaluations that were tested ($\Delta \bar{C_{f}}$, ${\Delta C}_{f_{w}}$ and $\Delta C_{f_{m}}$). From the results that were obtained, it was observed that Triple I showed the biggest average fruitlet capacitance differences mean $\bar{\Delta }C_{f}$ and had a smaller average coefficient of the variation $c_{v}$, when compared with Triple II.

Previous research studied the relative estimation of the water content for single flat-type air coil [23] that involved fruitlet capacitance, similar to this article. The coil structure that was used was the same as the one that was used in this research, only with a different number of turns *n* = 170. The difference between the estimated ripe and unripe capacitance value for the single flat-type air coil was 1.6483 pF. Even though it had a different number of turns, the estimated value could lie between *n* = 140 and *n* = 200. Regardless when comparing with triple series $\bar{\Delta }C_{f}$, the single coil’s capacitance difference was smaller than all of the three peaks of Triple I, however its capacitance was bigger than all of the Triple II peaks.

## 5. Conclusions

This paper covers a study about the triple flat-type coil series configuration for the oil palm fruit maturity sensor with two types of triple series, namely Triple I and II, with a constant number of turns (*n* = 200) and length (*l* = 5 mm), respectively. The performance of the first peak was highlighted in this study. From this study, it is observed that the resonance frequency increases with progressing ripening weeks, but that it is inversely proportional to the moisture content. However, the fruitlet capacitance decreases with the progressing ripening weeks and is directly proportional to the moisture content percentage.

For the triple series flat-type air coil peak resonance frequency evaluation, Triple I and II were compared peak-to-peak for the resonance frequency and fruitlet capacitance differences. When comparing the first peak of the Triple I and II resonance frequency mean difference $\bar{\Delta }f_{R}$, the Triple I has a smaller difference mean $\bar{\Delta }f_{R}$ = 169.12 kHz, in comparison with Triple II $\bar{\Delta }f_{R}$ = 226.54 kHz. For the second peak, even though the coil configuration for both of the triple series is the same, it seems that Triple I has a higher $\bar{\Delta }f_{R}$, as compared with Triple II, by 165.74 kHz. For the coefficient of the variation performance evaluation, the Triple II peaks have a rather high $c_{v}$ and therefore the level of dispersion around the difference mean is high, with an average of $c_{v}$ = 0.8401, as compared with the average $c_{v}$ for the Triple I peak of 0.2284. This shows that the Triple II is less precise compared to the Triple I for the resonance frequency difference comparison.

On the other hand, Triple I has a higher fruitlet capacitance differences mean $\bar{\Delta }C_{f}$, as compared with Triple II. When comparing the second peak of both of the triple series, the Triple I second peak is 5.25 times bigger, compared to the Triple II second peak, even though both have same *n* = 200 and *l* = 5 mm coil configuration. When comparing the Triple I and II average coefficient of variation $c_{v}$, Triple I has a smaller $c_{v}$ value when compared with Triple II, and is shown to be more precise as the lower value of the coefficient of variation produces a more precise estimated range of data.

In conclusion, the Triple I series coil with a fix number of turns (*n* = 200) with a different length shows better results compared to the Triple II coil with a fix length (*l* = 5 mm) and different number of turns. Triple I is more sensitive and precise compared with Triple II. The total length in the Triple I series allows more of the fruit surface area to touch the coil sensor and furthermore, it has less interwinding capacitance parameter intervention, as it has the same number of turns for all of the coil configurations in the series. Besides that, the Triple I and II sensor performance comparison using the resonance frequency and fruitlet capacitance showed an interesting trend, with both having their own unique points. The fruitlet capacitance is related to the permittivity changes as the fruit ripens, and the resonance frequency that is measured is used to calculate the fruitlet capacitance as well as to observe the shifting of resonance. The data from this study will help to decide on the best structure for the further improvement of the oil palm fruit ripeness stage detection, using an inductive-based method.

## Figures and Tables

**Table 1:** Type of coil configuration for triple series flat-type air coil sensor.

Type	Constant Parameter	First Coil Configuration	Second Coil Configuration	Third Coil Configuration
Triple I	*n =* 200	*l* = 10 mm	*l* = 5 mm	*l* = 3 mm
Triple II	*l* = 5 mm	*n* = 400	*n* = 200	*n* = 140

**Table 2:** Experiment setup parameters.

Parameter	Type/Value
Measurement setup	Series (*L_s_*–*R_s_*)
Voltage	500 mV
Frequency range	20–10 MHz
Points	200
Coil wire diameter	0.12 mm

**Table 3:** Triple series coil mean resonance frequency for the ripe and unripe, with difference between them.

Type	Peak	$\mathrm{Ripe}\;\mathrm{Mean},\;\bar{f_{Rr}}\;(\mathrm{MHz})$	$\mathrm{Unripe}\;\mathrm{Mean},\;\bar{f_{Ru}}\;(\mathrm{MHz})$	$\mathrm{Mean}\;\mathrm{Difference},\;\Delta \bar{f_{R}}\;(\mathrm{kHz})$
Triple I	First	2.980	2.849	130.66
	Second	4.437	4.181	256.28
	Third	6.915	6.643	271.35
Triple II	First	2.799	2.719	79.56
	Second	4.889	4.889	5.03
	Third	7.347	7.186	160.80

**Table 4:** Triple series coil αβ value for resonance frequency against the weeks and moisture graph.

Type	Peak	Week		Moisture Content	
		$\alpha_{wf_{R}}\;(\mathrm{Hz})$	$\beta_{wf_{R}}\;(\mathrm{Hz}/\mathrm{Week})$	$\alpha_{mf_{R}}\;(\mathrm{Hz})$	$\beta_{mf_{R}}\;(\mathrm{Hz}/\%)$
Triple I	First	2.81 × 10^6^	7.38 × 10^3^	3.03 × 10^6^	−2.14 × 10^3^
	Second	4.43 × 10^6^	11.90 × 10^3^	4.75 × 10^6^	−3.00 × 10^3^
	Third	6.55 × 10^6^	15.52 × 10^3^	7.05 × 10^6^	−5.26 × 10^3^
Triple II	First	2.51 × 10^6^	19.31 × 10^3^	2.83 × 10^6^	−1.75 × 10^3^
	Second	4.91 × 10^6^	6.20 × 10^3^	5.09 × 10^6^	−1.80 × 10^3^
	Third	7.12 × 10^6^	10.60 × 10^3^	7.37 × 10^6^	−2.16 × 10^3^

**Table 5:** Resonance frequency difference evaluation for triple series air coil.

Type	Peak	$\mathrm{Differences}\;\mathrm{Mean},\;\bar{\Delta }f_{R}\;(\mathrm{kHz})$	$\mathrm{Standard}\;\mathrm{Deviation},\;\sigma_{f}\;(\mathrm{kHz})$	$\mathrm{Coefficient}\;\mathrm{of}\;\mathrm{Variation},\;c_{v}\;$
Triple I	First	169.12	42.28	0.2500
	Second	272.86	24.08	0.0882
	Third	379.74	131.78	0.3470
Triple II	First	226.54	178.26	0.7869
	Second	107.12	91.07	0.8501
	Third	399.76	353.13	0.8834

**Table 6:** Triple coil mean fruitlet capacitance for ripe and unripe, with the difference between them.

Type	Peak	$\mathrm{Ripe}\;\mathrm{Mean},\;\bar{C_{fr}}\;(\mathrm{pF})$	$\mathrm{Unripe}\;\mathrm{Mean},\;\bar{C_{fu}}\;(\mathrm{pF})$	$\mathrm{Difference},\;\Delta \;\bar{C_{f}}\;(\mathrm{pF})$
Triple I	First	1.113	2.186	1.073
	Second	4.974	15.846	10.872
	Third	2.092	3.488	1.397
Triple II	First	0.147	0.395	0.247
	Second	1.471	2.032	0.561
	Third	0.356	0.452	0.095

**Table 7:** Triple coil αβ line fit value for fruitlet capacitance against the weeks and moisture graph.

Type	Peak	Week		Moisture Content	
		$\alpha_{wC_{f}}\;(F)$	$\alpha_{wC_{f}}\;(F/\mathrm{Week})$	$\alpha_{mC_{f}}\;(F)$	$\beta_{mC_{f}}\;(F/\%)$
Triple I	First	4.272 × 10^−12^	−193.301 × 10^−15^	0.141 × 10^−12^	32.314 × 10^−15^
	Second	8.454 × 10^−12^	−173.189 × 10^−15^	2.647 × 10^−12^	62.961 × 10^−15^
	Third	4.606 × 10^−12^	−139.312 × 10^−15^	1.475 × 10^−12^	25.313 × 10^−15^
Triple II	First	0.814 × 10^−12^	−25.878 × 10^−15^	0.374 × 10^−12^	2.666 × 10^−15^
	Second	2.562 × 10^−12^	−71.895 × 10^−15^	0.622 × 10^−12^	18.526 × 10^−15^
	Third	1.878 × 10^−12^	−13.404 × 10^−15^	1.006 × 10^−12^	11.739 × 10^−15^

**Table 8:** Fruitlet capacitance difference evaluation for triple series air coil.

Type	Peak	$\mathrm{Differences}\;\mathrm{Mean},\;\bar{\Delta }C_{f}\;(\mathrm{pF})$	$\mathrm{Standard}\;\mathrm{Deviation},\;\sigma_{c}\;(\mathrm{pF})$	$\mathrm{Coefficient}\;\mathrm{of}\;\mathrm{Variation},\;c_{v}$
Triple I	First	2.852	1.623	0.5692
	Second	6.993	3.582	0.5123
	Third	2.331	0.852	0.3654
Triple II	First	0.361	0.180	0.4998
	Second	1.332	0.681	0.5115
	Third	0.521	0.574	1.1010
